# Does the number of doses matter? A qualitative study of HPV vaccination acceptability nested in a dose reduction trial in Tanzania

**DOI:** 10.1016/j.tvr.2021.200217

**Published:** 2021-05-26

**Authors:** K.R. Mitchell, T. Erio, H.S. Whitworth, G. Marwerwe, J. Changalucha, K. Baisley, C.J. Lacey, R. Hayes, S. de SanJosé, D. Watson-Jones

**Affiliations:** a MRC/CSO Social and Public Health Sciences Unit, University of Glasgow, Berkeley Square, 99 Berkeley St, Glasgow, G3 7HR, UK; b Mwanza Intervention Trials Unit, National Institute of Medical Research, Isamilo, Mwanza, Tanzania; c Faculty of Epidemiology and Population Health, London School of Hygiene and Tropical Medicine, Keppel Street, London, WC1E 7HT, UK; d York Biomedical Research Institute & Hull York Medical School, University of York, John Hughlings Jackson Building, University Rd, Heslington, York YO10 5DD, UK; e Catalan Institute of Oncology, Avinguda de La Granvia de L’Hospitalet 199-203, 08908 L'Hospitalet de Llobregat (Barcelona), Spain; f Faculty of Infectious and Tropical Diseases, London School of Hygiene and Tropical Medicine, Keppel Street, London, WC1E 7HT, UK

**Keywords:** Acceptability, HPV vaccination, Qualitative, Dose reduction, Randomisation, Trial participation

## Abstract

**Background:**

The multi-dose regimen is a known barrier to successful human papillomavirus (HPV) vaccination. Emerging evidence suggests that one vaccine dose could protect against HPV. While there are clear advantages to a single dose schedule, beliefs about vaccine dosage in low and middle income countries (LMICs) are poorly understood. We investigated acceptability of dose-reduction among girls, and parents/guardians of girls, randomised to receive one, two or three doses in an HPV vaccine dose-reduction and immunobridging study (DoRIS trial) in Tanzania.

**Methods:**

Semi-structured interviews with girls (n = 19), and parents/guardians of girls (n = 18), enrolled in the study and completing their vaccine course.

**Results:**

Most participants said they entrusted decisions about the number of HPV vaccine doses to experts. Random allocation to the different dose groups did not feature highly in the decision to participate in the trial. Given a hypothetical choice, girls generally said they would prefer fewer doses in order to avoid the pain of injections. Parental views were mixed, with most wanting whichever dose was most efficacious. Nonetheless, a few parents equated a higher number of doses with greater protection.

**Conclusion:**

Vaccine trials and programmes will need to employ careful messaging to explain that one dose offers sufficient protection against HPV should emerging evidence from ongoing dose-reduction clinical trials support this.

## Highlights

• We interviewed girls, and parents/carers of girls, enrolled in an HPV Vaccine dose reduction trial.
• We found that enrolling in the trial in the context of community rumours required trust in the trial scientists.
• Scientists were trusted to decide on dosage; thus randomisation by dosage was not an acceptability issue.
• Girls preferred fewer vaccine doses in order to avoid injection-related pain.
• Parents/guardians generally wanted whichever dose regimen was most efficacious.

## 1 Introduction

Persistent human papillomavirus (HPV) infection, a pre-requisite for development of cervical cancer, can be prevented by three highly efficacious licensed vaccines that protect against infection with a number of high-risk HPV genotypes [[1], [10]]. The vaccine is usually targeted to girls prior to sexual debut and is currently administered in two or three dose schedules. As of May 2020, 127 countries across the world had introduced HPV vaccination programmes, but this comprised only 28% (22/78) of low-and-middle-income countries (LMIC) compared with 71% (105/147) of higher income nations [2]. There are stark global disparities, such that populations with the highest incidence of HPV infection and cervical cancer mortality are least likely to be protected by vaccination. By 2014, only 1% of women targeted by immunisation programmes globally were from low income countries [4].

Worldwide by 2014, 50.1% of the target population (typically girls between 9 and 15 years) had received one vaccination but only 39.7% had received the full course (minimum of two doses) [4]. Evidence from high income countries suggests that non-completion of the allocated dosing schedule is linked to ethnicity and healthcare coverage alongside other factors [5]. Number of doses is rarely investigated in studies of vaccine acceptability in low-income settings. However, in a small Kenyan study of women attending family planning services, more mothers said that they would be willing to have their daughters vaccinated with HPV vaccine if one injection was required (86%) compared with three (31%) [6]. Such preferences appeared underpinned by concerns about cost to families, an important barrier to acceptability in Sub-Saharan Africa [7]. A review of delivery strategies in LMIC concluded that non-vaccination and non-completion were driven more by programmatic factors, including lack of awareness and school absenteeism, than by opposition to the vaccine itself [8]. Specifically, in their review of health systems in LMIC, Wigle and colleagues highlighted the challenge of reaching girls with three doses of the vaccine where school attendance is very low [9].

A number of resource-poor countries have been hesitant in scaling-up HPV vaccination programmes, citing concerns about the sustainability of offering a multi-dose vaccine schedule to pre-adolescent and adolescent girls [10]. Feasibility of vaccine delivery is a key determinant of vaccine acceptability at national level [11] and the three-dose regimen is a known barrier to successful vaccination [12]. A reduction in the number of doses required is considered crucial to efforts to increase coverage in low income settings [4]. A single dose vaccination schedule would increase compliance, simplify delivery and allow considerable savings in terms of vaccines, consumables, disposal, outreach visit costs and staff time away from health facilities [13].

There is emerging biological evidence from studies conducted in Costa Rica [14] and India [15] that one dose could protect against HPV infection. If immune responses, efficacy and safety are confirmed to be similar following one dose compared to multi-dose schedules, adoption of a single dose regimen is likely to be rapid, particularly in resource-poor settings. Although the case for a single dose seems robust in terms of cost and accessibility, it cannot be simply assumed that one dose will be more acceptable to parents/guardians and their daughters. In Sub-Saharan Africa, vaccine uptake has tended to be high even where knowledge is low [7], but information is lacking on beliefs about dosage and whether these might have a bearing on vaccine acceptability. Prior to country programmes administering single-dose HPV vaccination, such questions are hypothetical, but they can be explored in more concrete ways among participants in current HPV dose-reduction trials.

We report views on dosage from a qualitative acceptability study conducted during the second year of an HPV vaccine trial (A Dose Reduction Immunobridging and Safety Study of two HPV vaccines in Tanzanian girls (DoRIS; clinicaltrials. gov: NCT02834637)) taking place in Mwanza, Tanzania [16].

## 2 Methods

### 2.1 Brief introduction to the parent trial

DoRIS is an unblinded, randomised trial whose main objectives are to compare the immunogenicity and safety of one, two and three doses of two different HPV vaccines in 9–14 year old Tanzanian girls [16,17]. After taking informed consent from parents/guardians and informed assent from potential participants, the trial enrolled 930 girls and randomly assigned them to one of 6 arms (155 girls per arm). Each arm received either the 2-valent (Cervarix®) or 9-valent (Gardasil-9®) vaccine, given as either one, two, or three dose schedules. Participants are being followed up for up to 60 months following the first dose. Blood is taken at specific follow-up visits for immunogenicity measurements, malaria testing and for antibodies to herpes simplex type 2 (HSV-2), a marker of sexual behaviour.

This paper presents findings of a nested qualitative acceptability study which aimed to understand attitudes of trial participants towards vaccine dosage and the HPV vaccine in general. Here we focus on objective one of the qualitative study: to explore views on vaccine dosage amongst trial participants and their parent/guardians, and whether these views have a bearing on acceptability of the vaccine.

### 2.2 Acceptability study design

We conducted semi-structured interviews with: i) girls aged 9 to 14 (n = 13), (ii) parents of (different) girls (n = 12) and (iii) girls and their parent/guardian in paired interviews (6 interviews). Originally the study planned to include interviews with individuals who did not complete their assigned vaccine schedule but remained in the trial. However, no eligible non-completers were identified in the trial (five girls (0.5%) missed one or two vaccine doses but left the study (e.g. due to relocation or concerns about vaccine safety); two girls (0.2%) missed their month 6 visit but were in the single dose arm and so did not miss a dose). Data for this qualitative study were collected between January and December 2018.

#### 2.2.1 Sampling

Girls who had completed their allocated vaccination course and attended their clinic visit scheduled for six months after the first vaccine dose, were eligible for selection. Girls were stratified by trial arm and age (9–11 or 12–14 years), and then selected for inclusion in the qualitative study using simple random sampling within strata. Random selection was done by the trial data manager using a computer randomisation algorithm. In each arm, 15 participants were selected (two back-up participants per interviewee in case of refusal). This list of 90 girls was then viewed by the research team to check for reasonable variation by religion, tribe, rural versus urban location, and primary versus secondary school. Parent/guardian interviewees were identified via daughters on this list.

#### 2.2.2 Participant recruitment

DoRIS trial staff members made initial contact with selected participants to introduce the acceptability study and to inform them that a member of the acceptability study team would be in touch with them. A female qualitative researcher followed up with each girl's parent/guardian to invite them to a brief introductory meeting to explain the study. This was held at a place convenient to participants (usually home; sometimes place of work). The researcher explained the study, indicated whether a girl, parent or paired interview was being requested, provided information sheets and addressed any questions. An interview date and place was scheduled a few days later with those expressing willingness to participate. If a parent or daughter declined to participate, a replacement was selected from the same stratum (this occurred only once). Parents provided written informed consent for themselves and on behalf of their daughters, depending on interview type. Illiterate parents provided witnessed consent by thumb print. Girls provided written assent prior to interview. Depending on participants' preferences, interviews were conducted at their home or at the study clinic. Choice of venue was based on convenience and need for privacy and quiet.

#### 2.2.3 Interview topic guide

The interviews probed for existing knowledge of the HPV vaccine and cancer; experience of getting the HPV vaccine (e.g. pain, side-effects; daughter's experience if parent was interviewed), views on vaccine dosage; and how the decision was made to take part in the DoRIS trial. The paired interviews explored parent-child dynamics as well as individual perspectives.

To facilitate discussion, participants were also presented with a set of cards, each describing a different factor that they may or may not have considered in deciding whether to participate in the trial. They were asked to sort the cards into three piles which reflected whether they had ‘thought about this a lot’, ‘thought about this a little or ‘did not consider this’. In paired interviews, parents and daughters sorted the cards together, with discussion. The cards included the following factors: severity of cervical cancer; risk of getting cervical cancer if not vaccinated; how effective the vaccine is; own age/daughters age; how safe the vaccine is; side-effects such as pain/fever; the fact that the vaccine protects against a disease that is sexually transmitted; and dosage. The card exercise was abandoned for younger girls (including some in paired interviews) because early interviewees found it too difficult.

#### 2.2.4 Analysis

All interviews were audio recorded with permission, transcribed verbatim into Swahili and then translated into English. Transcripts were checked for clarity and quality before and after translation. Data were analysed thematically following the Framework approach (Ritchie & Lewis, 2003) [18]. The analysis team (KM, TE and GM) first familiarized themselves with the data by reading through the English transcripts. An initial thematic coding frame was developed based on the study aims and initial reading of data. The coding frame was refined through reading of transcripts and discussion between analysts. All transcripts were read and coded by TE and GM in QSR NVivo 11 [19] using the final coding frame. The process was iterative and involved regular discussion between analysts, and going back and forth between data and interpretation. As a reliability check, a third of randomly selected transcripts were double coded and, via discussion, consistency across coders was deemed reasonable.

The card exercise served both as a discussion prompt and a means of ranking key factors based on how much they were considered in the decision to participate in the trial. Given the qualitative nature of the sample, these counts were simply intended to support and validate the qualitative insights [20].

### Ethical approval

The study was approved by the National Health Research Ethics Committee (NatHREC) in Tanzania (ref: NIMR/HQ/R.8a/Vol.IX/2682) and the Ethics Committee of the London School of Hygiene and Tropical Medicine (ref:11,972).

## 3 Results

### 3.1 Participants

The qualitative study participants were daughters and/or parents/guardians who had assented/consented to participate in the DoRIS trial and had completed their assigned vaccination schedule.

We conducted individual interviews with 13 girls and 12 (different) parents, plus interviews with 6 (different) girls together with their parent/guardian... All participants lived in the trial study location of Mwanza, a large city on the south-east edge of Lake Victoria. The sample was predominantly urban, Christian and educated to primary level (Table 1).

**Table 1:** Key characteristics of interview sample.

	GIRLS (n = 19)	PARENTS (n = 18)
Type of interview		
**Individual**	13	12
**Paired**	6	6
Age in years**, Median (range)**	12 (9–16)	44 (28–72)
Gender		
**Male**	0	4
**Female**	19	14
Residential setting		
**Urban**	15	17
**Peri-urban**	4	1
Religion		
**Christianity**	16	14
**Islam**	3	4
Current school level		
**Primary school**	12	–
**Secondary school**	7	–
Education Level of parent		
**Primary School**	–	14
**Secondary school**	–	2
**Vocational training**	–	1
**University**	–	1
Occupation of parent^a^		
**Vendor, salesman/woman**	–	6
**Farming, agricultural work**	–	4
**Housewife**	–	3
**Business man/woman**	–	3
**Unemployed**	–	1
**Other**	–	5
Number of HPV vaccine doses received	Girls	Daughters
		
**3 doses**	5	7
**2 doses**	7	5
**1 dose**	7	6

a Some parents gave more than one occupation.

### 3.2 Extent to which randomisation by dosage was considered in the decision to participate in the trial

All DoRIS trial participants were informed at the trial recruitment stage that they would be randomised to receive one, two or three vaccine doses. Among the participants in this qualitative study (all of whom had ultimately opted to participate in the trial), dosage did not appear to feature highly in their decision to participate.

**Fig. 1:**
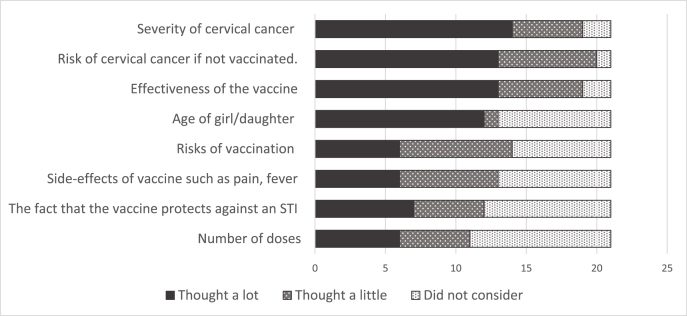
Card exercise showing extent to which factors were considered in decision to participate in trial.

As part of the interview, older girls (12–14 years), parents/guardians and parent/guardian-daughter dyads were asked to sort a set of cards listing potential considerations according to whether they thought about them a lot, a little or not at all (see methods and Fig. 1) when they were deciding whether or not to participate in the trial. Participants tended to think most about the consequences of getting cervical cancer, the risk of getting the disease if unvaccinated and the effectiveness of the vaccine:

*“Because I heard that when you get cervical cancer, it can lead you to death … and when I heard that there is a cancer vaccine […] I decided to take part.”* (Girl aged 13 years; 1 dose arm).

Appreciation of the severity of cervical cancer was sometimes based on personal experience:

*“because I have come across several cases … like two of them, people who suffered from cervical cancer, so when I saw that there is this vaccine and it can help … […] I saw it was good for [my daughter] to participate …*”*... (*Mother aged 45–50 years; 3 dose arm)

The next most common considerations were girl's age, vaccine safety, side-effects and the fact that the vaccine protected against an STI. It was notable that all the girls said they considered side-effects such as pain and fever, but half the parents said they did not consider this. While three-quarters of the parents thought a lot about whether their daughter was at an appropriate age for vaccination, only a third of girls did so (though note that small numbers mean these observations are only indicative).

Other considerations, particularly for parents, involved the cost of treating cervical cancer:

*“the first [consideration] was that I am poor. Because of poverty I will not be able to treat my child if she gets that disease because it is expensive to treat; what can I do? So I accepted [to join the trial].”* (Mother aged 45–50 years; 2 dose arm).

Similarly, the provision of free medical treatment during the trial was a consideration:

*“Even if I get ill, Dad simply calls them (research team) and I get fetched (by the researchers)."* (Girl aged 12 years; 3 dose arm)

### 3.3 Entrusting the dosage question to experts

Parents tended to frame the ultimate decision to participate in the trial as a matter of placing trust in the scientists and researchers. This trust in expertise in some ways simplified the decision to participate as it positioned key aspects of the trial – including dosage – as outside of their concern:

*“ ….about the number of doses, I did not consider that at all … because you (researchers) are the ones who know about [that]. I really don't know anything about [it], so I leave that to you guys to decide … If you tell me that within a year there is this number of doses, then it's okay … because I was the one who decided to take part […] We did not think at all about how many doses she would receive […] If you tell us 3 vaccine doses, or 2 or 1, that's ok.”* (Mother aged 45–50 years; 3 dose arm)

For the same reason, dosage did not appear to be a factor affecting the acceptability of the trial. For instance, the parent below was at pains to establish their lack of expertise while simultaneously demonstrating this lack through their misunderstanding of the vaccine as ‘treatment’:

*“ …. .… I am not the expert and the ones responsible for treating her are the experts who have studied this vaccine. So I may demand for her to be vaccinated with four doses which in turn may later be harmful yet the two doses could be enough to treat, so I cannot explain it in detail because I am not an expert “(*Mother aged 45–50 years; 2 dose arm)

This trust in experts with respect to dosage was illustrative of a broader trust, and even ‘faith’, in scientific research among those who had opted to participate in the trial:

*“My trust is through the seminars we received from the experts … because I am not an expert, I just agree with what I am told by them, as I said before I am doing this by faith […] they said it is a successful vaccine so I also trust it is going to be effective in her life.”* (Mother aged 35–40 years; 1 dose arm)

There was also a broader trust in government vaccination programmes, again sometimes borne of personal experience:

*“[ …] when I was growing up, so many people were dying of smallpox and a lot of children died due to measles but […] now even measles […] does [not] kill a lot of children and I can see that even smallpox has vanished … so that was something that was […] motivating me a lot … "* (Mother aged 50–55 years; 3 dose arm).

### 3.4 Trust and mistrust: the context of decision-making

The trust exhibited by those who had opted to join the trial may have reflected the typically polarised context in which the decision was made. Those attending the trial sensitisation meetings were sometimes required to weigh up information given by the trial implementers against opposition and rumours about vaccine trials circulating among neighbours and community members.

*“ … people were saying that white people intend to destroy our kids. Many parents had this same view; they'd asked the doctors about it but the doctors said it wasn't true and explained further. So some of them believed what doctors said but others quit [decided not to join] …. “(*Father aged 35–40 years; 2 dose arm)

These rumours were perceived as fear-inducing. They included that the vaccine would plant a bacteria in the body, sterilise their children or take away their virginity, and that vaccinations were part of a terrorist plot. Parents sometimes encountered these views immediately, on the walk home from sensitisation meetings:

*“[after the] parents meeting at school, we were returning home in groups […], they (neighbours/friends) asked us where we were coming from. We told them that we were called for cervical cancer vaccine meeting. Others said, ‘those things are nonsense. You are going to be planted with bacteria which will bring you problems in the future'."* Mother aged 40–45 years; 1 dose arm).

The decision to participate was complex and involved balancing benefit against risks that were difficult to assess without expert knowledge and training. Not having this knowledge themselves, community members ultimately had to decide whether to trust what they were told by the ‘doctors’ (trial implementers or other health professionals), draw on their own experience and knowledge, or listen to the beliefs circulating among the community. Participants described how they actively sought out advice from people they trusted, including family members and health professionals:

*“I went to consult that doctor (a friend). I told her that our children have been called at school for this [vaccine trial] and parents had also been informed. She told me that is fine, there is no problem ….so I became certain after talking to that doctor and I got peace.”* (Mother aged 40–45 years; 1 dose arm).

The sensitisation meetings and work by the trial team to explain the trial were fundamental in the decision:

*“ … but when they came and educated us and gave us the forms, we read and understood that this vaccine is very important and that is how I was courageous enough to let my daughter take part in the vaccination*” (Mother aged 50–55 years; 2 dose arm)

The parent above perceived the decision as one requiring courage. Fear of vaccination among community members and hesitancy about taking part, is illustrated in this account of parents changing their mind about participation after seeing that girls who were vaccinated were fine:

*“ … so later when they (parents who had previously refused to join the trial) saw that my daughter was doing well, they decided to […] ask […] me. I informed them that there were no more chances left.”* (Mother aged 45–50 years; 3 dose arm)

Against this backdrop of fear and hesitancy and having ultimately opted to put their faith in the scientists, the trust displayed by those taking part appeared to extend to all aspects of the trial, including the decisions around dose.

### 3.5 Hypothetical preferences for one, two or three doses

Both parents and daughters were asked whether, given a choice, they would prefer one, two or three doses of the vaccine. Some (particularly the girls) found this hypothetical question difficult; they were able to state a preference but not always able to give a reason. Some found it difficult to think of advantages of one dose schedule over another. Others, particularly parents, were reluctant to give an opinion, believing that it was a matter of scientific research. A few girls were confused between dosage and trial randomisation arm, assuming that arm A must be the ‘best’ since A is associated with a top grade at school. Several parents misunderstood or forgot the randomisation by dosage, believing allocation to be about child age, weight or degree of sexual risk. Among those who did express views on dosage, four considerations emerged: avoidance of pain, societal benefit, cost saving, and efficacy.

*Pain avoidance.* Almost all of the girls said they would prefer one dose of the vaccine, rather than two or three. This was primarily due to fear of the injection and a desire to avoid the pain it caused:

*‘I love the vaccination but I am afraid […] I feel like the syringe is too long and I can't imagine that all of it enters my body’* (Girl aged 10 years; 2 dose arm).

Most girls expressing a preference to avoid pain did so on the understanding that one dose was just as likely to be efficacious as two or three. However, for some girls fear of the injection over-rode any concerns about which number of doses was most effective; they would prefer one dose regardless. Girls in the one dose arms reported feeling ‘lucky’ and happy with their group since they had received fewer injections than girls in other arms. A few girls who had received three doses reported negative reactions from those receiving one or two doses, although this did not deter them from participation:

*“From the whole group, I was the only one who was given 3 doses, they (other students) asked me, ‘you mean they are injecting you three times?! … We are being injected only once. It is better if you just quit'[…] I just ignored them”,* (Girl aged 14 years (paired interview); 3 dose arm*).*

*Societal benefit:* Four parents expressed a preference for one dose on the basis that one dose per child would mean the vaccine could be distributed among far more children for the same cost:

*“Well, I would wish that my daughter would have been given one vaccine dose so that others also benefit. It will spread among people […] It means that for that girl who had received two vaccine doses, then two children would have been vaccinated”* (Mother aged 50–55 years; 3 dose arm).

*Reduced cost:* A few parents also raised the point that outside of the trial context, one dose would imply less personal cost. In addition to payment for the vaccine itself, they mentioned the cost of travelling to a clinic and potential lost earnings involved in accompanying children. Asked why they thought the scientists might be investigating whether one dose could be effective, one parent thought it could be about broader cost savings:

*“Perhaps, they (researchers) have seen that one vaccine dose has the same protection and that is why they see a possibility of reducing [it]. They [the trial scientists] mentioned that these vaccine doses are very expensive, so probably to also reduce cost”.* (Mother aged 35–40 years; 1 dose arm).

Conversely, in the context of the vaccine trial, three doses were seen as beneficial by some, because they implied more clinical attention to their daughter and this was viewed as a benefit:

*“I really wished [my daughter] had received three vaccine doses. […] Yes, there is an extra thing (in three doses) because they have researched on her more.* (Mother aged 45–50 years; 2 dose arm).

*Efficacy:* Most parents recalled that the trial sought to establish whether different numbers of doses might be equally efficacious. As described above, the majority felt efficacy was ultimately a matter for the scientists, and none of the participants expressed a strong preference for a particular dose based on efficacy. A few parents and girls thought that two or three doses might offer more protection and would be more ‘helpful’ (compared with one dose). There was also a perception that two or three doses would stay in the body longer, offering a longer period of protection. One parent thought that three doses were each a third as ‘strong’ as one dose, and another expressed concern therefore that one dose might be ‘*too much medicine’* in one go (mother age 50–55 years; 3 dose arm).

One parent whose daughter received two doses said she wished *‘that if there is a possibility of adding some more, then give her that remaining one so that she can finish’ (*Father aged 45–50 years; 2 dose arm), implying a belief that a girl receiving less than three doses had not received the full course. Belief that three doses offered more protection was also implied in the view that if a girl is sexually active, she may require more doses*:*

*“Because I see [HPV] is a result of sex. It means if she likes [sex] so much, it's better to receive two vaccines”* (Mother aged 40–45 years; 1 dose arm).

Of girls expressing an opinion on efficacy, several thought that the doses were equivalent:

“*that one dose is as powerful as the other doses”*. (Girl aged 14 years: 1 dose).

Only a few girls said they thought that two/three doses might be more efficacious than one dose. These girls tended to view two and three doses as equally effective and generally said that they would prefer two doses because it implied one less injection.

## 4 Discussion

This study explored whether dosage mattered to girls, and parents/guardians of girls, who had enrolled in an HPV vaccine dose reduction trial and completed all of their scheduled doses. We found that being randomly allocated to the number of doses did not feature highly in their decision to participate. Instead participants thought primarily about the consequences of getting cervical cancer, the risk of getting it if unvaccinated and the effectiveness of the vaccine. Girls were particularly concerned about the pain of injection. In the context of the trial, decisions about dosage were viewed as outside of participant expertise and therefore not their concern. Entrusting this to experts reflected, to some extent, the polarised context in which the decision to participate in the trial was enacted. Having ultimately opted to put their faith in the scientists, the trust displayed by those taking part appeared to extend to all aspects of the trial, including randomisation by dose. Given a hypothetical choice, girls generally said that they would prefer fewer doses in order to avoid the pain of injections. Views among parents were mixed, with most wanting whichever dose was most efficacious. Nonetheless, a few parents equated a higher number of doses with greater protection, believing that the vaccine would stay longer in the body or that three injections implied the full course. In the context of a trial, three doses was also perceived as implying more medical attention to an enrolled daughter.

This study is rare in its explicit focus on dosage in relation to HPV vaccine acceptability and in directly seeking the views of girls as well as their parents. It contributes to a sparse literature on actual HPV vaccine acceptability in low-income settings. The main limitation is that the sample comprises only girls, and/or parents/guardians of girls, who agreed to participation in a vaccine trial and who completed their clinic visit scheduled for six months after the first vaccine dose. Although we originally planned to interview girls, and parents of girls, who did not complete their vaccine course, no girls met the study protocol criteria for non-completion of the vaccine schedule (i.e. missing one or more vaccine doses) but remaining in the study. Five girls (0.5%) missed one or two vaccine doses but left the study (e.g. due to relocation); two girls (0.2%) missed their month 6 visit but were in the single dose arm and so did not miss a dose. The absence of non-completion in itself provides further support for the qualitative evidence suggesting that dosage randomisation did not have a bearing on the acceptability of the trial among those opting to enrol in it. However, we do not know whether other families or girls declined participation because of concerns related to vaccine dosage. The active community liaison system for the trial had not detected any negative rumours related to vaccine dosage at the completion of the acceptability study, but it is possible that we missed rumours circulating among the wider community. It is also worth noting that the DoRIS trial did not have a placebo control arm and this may have led to enrolment of a group more positively disposed to the HPV vaccine (since getting the vaccine was certain). Some of the positivity towards the HPV vaccine may have reflected an element of resoluteness in the context of perceived opposition from neighbours. Compared with the rumours around vaccine safety, uncertainty around dosage may have appeared a risk of much lower magnitude. It is difficult to determine whether, outside of a trial context, dosage would remain of small significance.

In terms of the qualitative design, we opted to include paired interviews because we were interested in whether the dynamic between parent/guardian and child could provide insight into decision making processes. What we actually found was that daughter participants tended to say less and defer to their parent/guardian, such that these interviews were less good at drawing out daughter views. Finally, in common with all qualitative research, the study provides a nuanced account of the range of views and experiences, but cannot comment on their frequency or associations with other factors of interest.

The girls and parents interviewed in this study often struggled with the concept of randomisation by dosage. Difficulty understanding the concept of randomisation has also been identified among adolescents (16–19 years) in the context of HIV vaccine trials in high-income country settings [21,22]. Communicating risk is complicated [23]; key questions regard the right level of information, how to couch uncertainty and the risk of introducing doubt or legitimising rumours by attempting to counter them. In the context of African vaccine trials, it is suggested that limited knowledge can elevate concerns among participants [[24], [25], [26], [27]]. However, retention in the DoRIS trial has been very high with >95% attending their clinic visit scheduled for 24 months after the first vaccine dose. This suggests that participants do value their ongoing participation in the trial.

We found a tendency among a few parents/guardians to equate a higher number of doses with greater protection. Any national vaccination programme switching to a lower dose will need to consider how to address this belief. Since fewer doses are also recognised to be cheaper, there may also be suspicion that a switch to fewer doses is driven by a desire (by government or vaccinators) to cut costs. These views may be counterbalanced by the greater convenience (and in some cases cost-saving) to participants of a single dose, and for girls, the benefit of fewer injections. Any education strategies designed to manage a switch to a lower dose regimen could incorporate these findings into their messaging. Vaccination programmes over many decades have shown that strategies beyond information-giving are also required to support and facilitate implicit trust in the science of vaccination [28]. Strategies such as community engagement are essential but cannot necessarily prevent opposition and there is tension between recruiting sufficient numbers for trials and empowering communities to potentially say ‘no’ to participation [29].

## 5 Conclusions

A switch to a single-dose vaccine will alleviate some but not all of the barriers to vaccine coverage. In settings where the vaccine is administered in primary care, it will remove the need for additional visits [30] and the risk that subsequent doses will be forgotten [31]. It will also significantly reduce overall delivery costs and may make it easier to integrate a single vaccination visit with other school-based health interventions. Our study of HPV trial participants suggests that switching to a single-dose vaccine would not pose major acceptability issues among those pre-disposed to vaccine uptake. Given the misunderstandings highlighted in our study, vaccine trials and programmes will need to employ careful messaging to highlight the benefits of a single dose and to explain that one dose offers sufficient protection against HPV.

In addition, other system-level barriers common to many resource-poor settings - inadequate infrastructure, limited heath professional training, lack of means to pay, lack of a regular health care provider, inconsistent endorsement of vaccine by health care providers, little contact with medical system; low school attendance [32,33] - will still need to be tackled and efforts to address them maintained. Since completion of this study, global vaccination efforts to combat the spread of Cov-Sars-2 have drawn renewed attention to vaccine hesitancy and barriers to implementation of programmes, and will undoubtedly have a bearing on how these challenges are tackled in future.

## FUNDING sources

This work was supported by the 10.13039/501100000265Medical Research Council [MR/N006135/1]; the 10.13039/100000865Bill and Melinda Gates Foundation [OPP1167526]. KRM is supported by the 10.13039/501100000589Chief Scientist Office (Scotland) [SPHSU11; SPHSU18] and UK 10.13039/501100000265Medical Research Council [MC_UU_1,201,711; MC_UU_00022/3]. RH and KB receive funding from the UK 10.13039/501100000265Medical Research Council (10.13039/501100000265MRC) and the UK 10.13039/501100002992Department for International Development (DFID) under the 10.13039/501100000265MRC/DFID Concordat agreement, which is also part of the EDCTP2 programme supported by the 10.13039/501100000780European Union [Grant Ref: MR/R010161/1].

## Authorship statement

DWJ: conceived and designed the study, KB: conceived and designed the study, HW: conceived and designed the study, TE: undertook the interviews, GM: undertook the interviews, KM undertook analysis and early drafting of result, wrote the manuscript with critical intellectual input, and approval to submit, from all authors, JC: gave scientific input into analysis and interpretation of the data, CL: gave scientific input into analysis and interpretation of the data, RH: gave scientific input into analysis and interpretation of the data, SS: gave scientific input into analysis and interpretation of the data. All authors reviewed and approved the final version of the manuscript.

## Declaration of competing interest

The authors declare that they have no known competing financial interests or personal relationships that could have appeared to influence the work reported in this paper.

## References

[1] SAGE (2018). Meeting of the strategic advisory group of experts on immunization, october 2018 - conclusions and recommendations. Wkly. Epidemiol. Rec..

[2] PATH (2020). Global HPV Vaccine Introduction Overview. https://path.azureedge.net/media/documents/Global_HPV_Vaccine_Intro_Overview_Slides_webversion_2020May.pdf.

[4] Bruni L., Diaz M., Barrionuevo-Rosas L., Herrero R., Bray F., Bosch F.X., de Sanjosé S., Castellsagué X. (2016). Global estimates of human papillomavirus vaccination coverage by region and income level: a pooled analysis. The Lancet Global Health.

[5] Kessels S.J., Marshall H.S., Watson M., Braunack-Mayer A.J., Reuzel R., Tooher R.L. (2012). Factors associated with HPV vaccine uptake in teenage girls: a systematic review. Vaccine.

[6] Becker-Dreps S., Otieno W.A., Brewer N.T., Agot K., Smith J.S. (2010). HPV vaccine acceptability among Kenyan women. Vaccine.

[7] Cunningham M.S., Davison C., Aronson K.J. (2014). HPV vaccine acceptability in Africa: a systematic review. Prev. Med..

[8] LaMontagne D.S., Barge S., Thi Le N., Mugisha E., Penny M.E., Gandhi S., Janmohamed A., Kumakech E., Mosqueira N.R., Nguyen N.Q. (2011). Human papillomavirus vaccine delivery strategies that achieved high coverage in low-and middle-income countries. Bull. World Health Organ..

[9] Wigle J., Coast E., Watson-Jones D. (2013). Human papillomavirus (HPV) vaccine implementation in low and middle-income countries (LMICs): health system experiences and prospects. Vaccine.

[10] Gallagher K.E., LaMontagne D.S., Watson-Jones D. (2018). Status of HPV vaccine introduction and barriers to country uptake. Vaccine.

[11] Winkler J.L., Wittet S., Bartolini R.M., Creed-Kanashiro H.M., Lazcano-Ponce E., Lewis-Bell K., Lewis M.J., Penny M.E. (2008). Determinants of human papillomavirus vaccine acceptability in Latin America and the Caribbean. Vaccine.

[12] Walling E.B., Benzoni N., Dornfeld J., Bhandari R., Sisk B.A., Garbutt J., Colditz G. (2016). Interventions to improve HPV vaccine uptake: a systematic review. Pediatrics.

[13] Lazcano-Ponce E., Stanley M., Muñoz N., Torres L., Cruz-Valdez A., Salmerón J., Rojas R., Herrero R., Hernández-Ávila M. (2014). Overcoming barriers to HPV vaccination: non-inferiority of antibody response to human papillomavirus 16/18 vaccine in adolescents vaccinated with a two-dose vs. a three-dose schedule at 21 months. Vaccine.

[14] Kreimer A.R., Herrero R., Sampson J.N., Porras C., Lowy D.R., Schiller J.T., Schiffman M., Rodriguez A.C., Chanock S., Jimenez S. (2018). Evidence for single-dose protection by the bivalent HPV vaccine—review of the Costa Rica HPV vaccine trial and future research studies. Vaccine.

[15] Sankaranarayanan R., Joshi S., Muwonge R., Esmy P.O., Basu P., Prabhu P., Bhatla N., Nene B.M., Shaw J., Poli U.R.R. (2018). Can a single dose of human papillomavirus (HPV) vaccine prevent cervical cancer? Early findings from an Indian study. Vaccine.

[16] Baisley K.J., Whitworth H.S., Changalucha J., Pinto L., Dillner J., Kapiga S., de Sanjosé S., Mayaud P., Hayes R.J., Lacey C.J. (2021). A dose-reduction HPV vaccine immunobridging trial of two HPV vaccines among adolescent girls in Tanzania (the DoRIS trial) - study protocol for a randomised controlled trial. Contemp. Clin. Trials.

[17] A Dose Reduction Immunobridging and Safety Study of Two Human Papillomavirus (HPV) Vaccines in Tanzanian Girls (DoRIS): Enrollment and Progress to Date. https://clinicaltrials.gov/ct2/show/NCT02834637.

[18] Ritchie J., Lewis J., McNaughton Nicholls C., Ormston R. (2003). Qualitative Research Practice: A Guide for Social Science Students and Researchers.

[19] Ltd. QIP (2015). NVivo Qualitative Analysis Software. Version 11.

[20] Sandelowski M. (2001). Real qualitative researchers do not count: the use of numbers in qualitative research. Res. Nurs. Health.

[21] Blake D.R., Lemay C.A., Kearney M.H., Mazor K.M. (2011). Adolescents' understanding of research concepts: a focus group study. Arch. Pediatr. Adolesc. Med..

[22] Ott M.A., Alexander A.B., Lally M., Steever J.B., Zimet G.D. (2013). Interventions AMTNfHA: preventive misconception and adolescents’ knowledge about HIV vaccine trials. J. Med. Ethics.

[23] Zimet G.D., Rosberger Z., Fisher W.A., Perez S., Stupiansky N.W. (2013). Beliefs, behaviors and HPV vaccine: correcting the myths and the misinformation. Prev. Med..

[24] Jaoko W., Nakwagala F.N., Anzala O., Manyonyi G.O., Birungi J., Nanvubya A., Bashir F., Bhatt K., Ogutu H., Wakasiaka S. (2008). Safety and immunogenicity of recombinant low-dosage HIV-1 A vaccine candidates vectored by plasmid pTHr DNA or modified vaccinia virus Ankara (MVA) in humans in East Africa. Vaccine.

[25] Kibuuka H., Guwatudde D., Kimutai R., Maganga L., Maboko L., Watyema C., Sawe F., Shaffer D., Matsiko D., Millard M. (2009). Contraceptive use in women enrolled into preventive HIV vaccine trials: experience from a phase I/II trial in East Africa. PloS One.

[26] Mugerwa R.D., Kaleebu P., Mugyenyi P., Katongole-Mbidde E., Hom D.L., Byaruhanga R., Salata R.A., Ellner J.J. (2002). First trial of the HIV-1 vaccine in Africa: Ugandan experience. Br. Med. J..

[27] Omosa-Manyonyi G.S., Jaoko W., Anzala O., Ogutu H., Wakasiaka S., Malogo R., Nyange J., Njuguna P., Ndinya-Achola J., Bhatt K. (2011). Reasons for ineligibility in phase 1 and 2A HIV vaccine clinical trials at Kenya AIDS vaccine initiative (KAVI), Kenya. PloS One.

[28] Herzog T.J., Huh W.K., Downs L.S., Smith J.S., Bj M. (2008). Initial lessons learned in HPV vaccination. Gynecol. Oncol..

[29] Swartz L., Kagee A. (2006). Community participation in AIDS vaccine trials: empowerment or science?. Soc. Sci. Med..

[30] Rand C.M., Szilagyi P.G., Albertin C., Auinger P. (2007). Additional health care visits needed among adolescents for human papillomavirus vaccine delivery within medical homes: a national study. Pediatrics.

[31] Dorell C.G., Yankey D., Santibanez T.A., Markowitz L.E. (2011). Human papillomavirus vaccination series initiation and completion. Pediatrics.

[32] Black E., Richmond R. (2018). Prevention of cervical cancer in Sub-Saharan Africa: the advantages and challenges of HPV vaccination. Vaccines.

[33] Sankaranarayanan R., Anorlu R., Sangwa-Lugoma G., Denny L.A. (2013). Infrastructure requirements for human papillomavirus vaccination and cervical cancer screening in sub-Saharan Africa. Vaccine.

